# Anemia among Women Who Visit Bost Hospital for Delivery in Helmand Province, Afghanistan

**DOI:** 10.1155/2021/9358464

**Published:** 2021-01-05

**Authors:** Zabihullah Anwary, Muhammad Haroon Stanikzai, Wali Mohammad Wyar, Abdul Wahed Wasiq, Khushhal Farooqi

**Affiliations:** ^1^Faculty of Medicine, Bost University, Lashkar Gah, Helmand, Afghanistan; ^2^Public Health Department, Faculty of Medicine, Kandahar University, Kandahar, Afghanistan; ^3^Para Clinic Department, Faculty of Medicine, Kandahar University, Kandahar, Afghanistan; ^4^Internal Medicine Department, Faculty of Medicine, Kandahar University, Kandahar, Afghanistan; ^5^Pediatrics Department, Faculty of Medicine, Kandahar University, Kandahar, Afghanistan

## Abstract

**Background:**

Anemia is a global public health problem that affects a large number of pregnant women worldwide. In developed and developing countries, the number of pregnant women who become anemic ranges between 18% and 56%, respectively. The aim of this study was to determine the prevalence of anemia and factors associated with anemia among pregnant women who visit Bost Hospital for delivery in Helmand province, Afghanistan.

**Methods:**

This was a hospital-based cross-sectional study that included 787 pregnant women who visited Bost Hospital for delivery services from January to June 2019. Data was collected in a self-structured questionnaire, which included sociodemographic, obstetrics, and laboratory information. Data was analyzed using SPSS 21.00 Statistical software. The prevalence of anemia was presented as a percentage. Bivariate analysis and binary logistic regression were used to identify the predictors of anemia among pregnant women.

**Results:**

The overall prevalence of anemia in this study was 51% (95% CI = 48.7%–54.7%). The mean hemoglobin concentration among the study participants was 10.8 (±1.8) g/dL. On bivariate analysis, age group 30 years and above, rural residency and unemployment/housewives, multiparity, and no previous use of contraceptive were found to be associated with anemia. Binary logistic regression showed that multiparity (AOR = 3.09, 95% CI = 1.81–5.29) and no contraceptive use (AOR = 1.53, 95% CI = 1.08–2.16) were the independent predictors of increased anemia among pregnant women.

**Conclusion:**

Anemia was found to be a severe public health problem in the study area. Policymakers in Afghanistan must accelerate interventions to promote family planning. The need for prospective studies is also suggested to identify other factors associated with anemia among pregnant women.

## 1. Background

Anemia among pregnant women has proven to be a global public health problem, mainly affecting low- and middle-income countries. It is defined by the World Health Organization (WHO) as a hemoglobin level less than 11gr/dl at any time during pregnancy [1]. It is classified as severe, moderate, and mild when concentrations of hemoglobin (Hb) are <7 g/dL, 7–9.9 g/dL, and 10-11 g/dL, respectively [1, 2].

Anemia can affect anyone at any stage of life. However, it mainly affects pregnant women and children under the age of five. Globally, it affected more than 1.62 billion (25%) people; of these, 56 million cases were among pregnant women [3, 4]. The prevalence of anemia among pregnant women varies in different parts of the world. For instance, its global prevalence is estimated at 41.8%, with the highest prevalence in sub-Saharan Africa (61.3%) [5, 6]. Due to the conflict and instability in Afghanistan, anemia among pregnant women has been less researched. WHO publications and Demographic Health Surveys (DHS) have summarized that anemia is prevalent in 38.2 % of cases among pregnant women in Afghanistan [5].

Anemia among women who are pregnant has significant adverse health effects. It can deteriorate women's health and raise neonates' risk of adverse health outcomes [7]. In addition, several studies indicated that low-birth-weight, preterm birth, infection, and hemorrhage are caused by anemia during pregnancy [7–9]. WHO recommends full blood count testing for the diagnosis of anemia during pregnancy. However, on-site hemoglobin testing with a portable hemoglobinometer is faster, simpler, and less expensive, and hence, it is recommended for diagnosing anemia in settings where full blood count testing is not available [10].

Various studies have reported anemia among pregnant women [1, 5, 11–19]. In developed and developing countries, the proportion of pregnant women who become anemic varies from 18% to 56%, respectively [5, 18]. Maternal age, marital age, gestational age, residency, gravidity, parity, maternal level of education, inadequate access to iron/folic supplements, and insufficient birth spacing have been found to be associated with anemia among pregnant women [1–3, 8–19]. Evaluation of anemia status and understanding of the factors associated with anemia among pregnant women is critical in reassuring maternal health as well as neonatal health. Monitoring of the Hb level is very important to treat and prevent anemia. Few studies reported in Afghanistan on anemia among pregnant women. Therefore, this study aimed to assess the proportion of anemia and identify factors that are associated with anemia among pregnant women who visit Bost Hospital for delivery in Helmand province.

## 2. Methods

### 2.1. Study Setting and Period

This facility-based study was conducted in Bost Hospital of Helmand province, Afghanistan, from January to June 2019. Bost Hospital, a 300-bed facility run by the Ministry of Public Health (MoPH) in collaboration with the Médecins Sans Frontières (MSF), is located at the center of Lashkargah city. This provincial hospital provides a variety of curative and preventive services with special emphasis on curative services to approximately 500000 urban and rural populations.

### 2.2. Study Design and Population

This study was based on cross-sectional data analysis. Study participants were pregnant women who visited Bost Hospital for delivery in Helmand province. According to WHO recommendations, pregnant women with hemoglobin values below 11 g/dl were considered anemic.

Inclusion and exclusion criteria were as follows: pregnant women who visited Bost Hospital for delivery during the study period were included. Pregnant women with antepartum hemorrhage, who recently transfused blood, who receive therapy for anemia, and who were unable to respond due to severe illness or unwillingness to participate in the study were excluded.

### 2.3. Sample Size and Sampling Procedures

A total of 787 pregnant women were included in the study. The sample size was determined using the single population proportion's general formula, with the following assumptions: prevalence of anemia in the study area (P) of 38.5% [5], confidence level (CI) of 95%, and margin of error of 5%. We were given a sample size of 363 by this estimate. For the accuracy and validity of the analysis, the sample size was increased to 787.

With regard to the sampling methods, we consecutively included all pregnant women who visited Bost Hospital for delivery services during the study period.

### 2.4. Data Collection Methods

A self-structured and pretested questionnaire was used to obtain study participants' sociodemographic information, history of obstetrics, and level of Hb. The questionnaire was first prepared in English; later, it was translated to Pashtu and back to English in order to obtain the validity of contents. A face-to-face interview was used to collect data. As data collectors, three clinical nurses, one laboratory technician, and one supervisor were involved. In the health facility, suspected cases of anemia were subjected to Hb level measurement. However, we measured the Hb level of all pregnant women during the study period using the portable device Acon Mission Plus HB meter. As per WHO recommendations, pregnant women with hemoglobin values below 11 g/dl were considered anemic and they were classified as mild (10–10.9 g/dl), moderate (7–9.9 g/dl), severe (below 7 g/dl), and very severe anemic (below 4 g/dl). [1].

### 2.5. Data Analysis

Collected data were coded and entered into Microsoft Excel (2016). Data quality was checked for consistency, completeness, and accuracy. The data was analyzed using IBM SPSS version 21 [20]. Prevalence of anemia among pregnant women was presented as percentages. Bivariate analysis was used for the possible factors associated with anemia. To determine the strength of association, the Odds Ratio (Chi-square test, Mantel–Haenszel statistics) was estimated. Stepwise multiple logistic regression (forward LR method) was carried out to identify independent determinants for anemia. The factors, which had an association with anemia on bivariate analysis, were included in the multiple regression analysis.

### 2.6. Ethical Consideration

Ethical approval of the study was obtained from the research committee of the Medical Faculty, Kandahar University. This research has also been approved by the Helmand Public Health Directorate. Informed consent was sought from all study participants. To maintain the confidentiality of the participants in the study, unique identifiers were removed from the data analysis.

## 3. Results

A total of 787 pregnant women who visited Bost Hospital for delivery were included in the analysis. The mean age of pregnant women was 30.48 years (±7.02). Of the total, 61.4% were of age above 30 years and 64.4% belonged to a rural residence. More than half of the pregnant women (53.3%) had no formal education and 93.8 % were housewives. With reference to the education of the husband, 51.7% of them were uneducated. Over half of households (53.3%) had monthly income ranging from 5000 to 10000. 42.4% and 3.3% of households had >10000 and <5000 monthly incomes, respectively, Table 1 shows sociodemographic information of study participants at baseline.

All pregnant women who participated in this study were presented in the third trimester. Most of the pregnant women (89.1%) had single parity. Approximately 70.1% of women in the past had not used contraception. With reference to the birth interval, most study participants (93.3%) had no birth interval information. In just nine cases, more than two years of the birth interval was documented, while 44 cases had less than two years of birth interval.  Table 2 shows obstetrics and medical-related characteristics of study participants at baseline.

**Figure 1:**
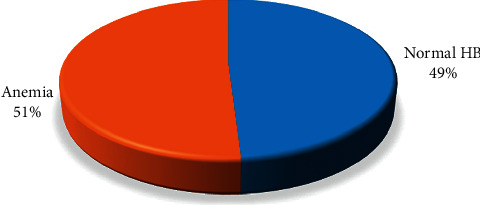
Prevalence of anemia among pregnant women who visit Bost Hospital for delivery (*n* = 787).

**Figure 2:**
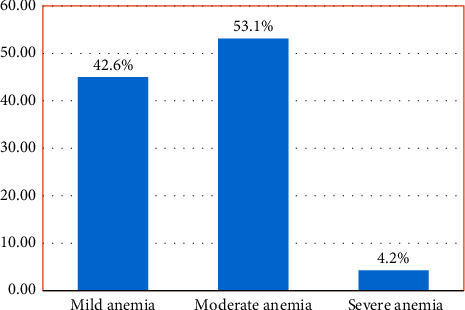
Percentage of anemia by severity among anemic pregnant women (*n* = 401).

The overall prevalence of anemia in this study was 51% (95% CI = 48.7%–54.7%) (Figure 1). The mean (SD) hemoglobin concentration among the study participants was 10.8 (±1.8) g/dL. Of the anemic pregnant women, 171 (42.6%), 213(53.1%), and 17 (4.2%) had mild anemia (Hb ranges 10.0–10.9 g/dL), moderate anemia (Hb ranges 7.0–9.9 g/dL), and severe anemia (Hb < 7.0 g/dL), respectively (Figure 2).

Bivariate analysis of the factors associated with anemia among pregnant women is shown in Table 3. Age 30 years and above (*p*=0.002, COR = 1.19), rural residency (*p*=0.002, COR = 1.71), unemployment/housewives (*p*=0.017, COR = 1.04), multiparity (*p* ≤ 0.0001, OR = 2.80), and no previous use of contraceptives were significantly associated with anemia. These factors were included in the binary logistic regression model. It was found that multiparity (AOR = 3.09, 95% CI = 1.81–5.29) and no previous uses of contraceptives (AOR = 1.53, 95% CI = 1.08–2.16) were significantly associated with anemia.

## 4. Discussion

This study was carried out to determine the prevalence of anemia and identify factors that are associated with anemia among pregnant women who attend Bost Hospital for delivery in the Helmand province of Afghanistan. Monitoring of hemoglobin level provides very important inputs for preventing and treating anemia among pregnant women. It is of particular importance in countries like Afghanistan that have been affected by the high Maternal Mortality Ratio (MMR) and conflict-affected weak health systems.

In this study, the proportions of pregnant women who become anemic were 51%. According to the WHO classification of public health significance of anemia, the severity indicates that it is a severe public health problem among pregnant women in the study area [5]. Studies from different developing countries found that 15–59% of pregnant women suffer from anemia during pregnancy [11–18]. However, the prevalence of anemia in this study was higher than that in other studies conducted in Afghanistan [5]. This may be an overestimation of actual figures in this study as this population is only pregnant women who visit the hospital for delivery, which is dominated by those who are in the third trimester. According to published literature, anemia among pregnant women was significantly associated with the third trimester of gestational age [1, 3, 11, 13–16]. Although the prevalence of anemia, in this study, was lower than that in other studies conducted in Pakistan (57.7%) [11] and Sudan (53%) [16], it was higher than that in Iran (16.8 %) [12], India (33.9%) [1], and Bangladesh (34.7%) [13]. Variation in prevalence of anemia reported in different studies can be attributed to variation in socioeconomic status, geographic locations, research methodology, dietary habits of study participants, and other nonexplored factors.

This study revealed that multiple parities (AOR = 3.09, 95% CI = 1.81–5.29) and no previous use of contraceptives (AOR = 1.53, 95% CI = 1.08–2.16) were the independent predictors of anemia among pregnant women. Multiparity and no previous use of contraceptives were documented as important risk factors of anemia among pregnant women in the literature. Several studies have found multiple parities and no previous use of contraceptives as important predictors of anemia among pregnant women (Ethiopia, Ghana, and India—multiparity [14, 15, 19], and Tanzania and Nepal—no previous use of contraceptives [22, 23]). Consistent with previous studies, every pregnancy can increase the risk of hemorrhage before, during, and after pregnancy. Hence, multiparty aggravates the risk of hemorrhage. Furthermore, iron and other nutrients are depleted during increased and repeated pregnancies. The use of contraceptives not only reduces the number of parities but also has noncontraceptive benefits. The use of contraception as protection against anemia has been documented in several studies. Women with multiparity and no previous use of contraceptives are at higher risk of anemia, develop maternal complications, and are associated with higher adverse health outcomes in neonates [7–9]. Hence, all families should be educated on the importance of family planning at the earliest to avoid anemia during pregnancy.

In addition to multiparity and no previous use of contraceptives, we also identified an association between anemia and old age, rural residency, and unemployment. These factors did not achieve significance in binary logistic regression. Many studies in the past have found that anemia was higher in women of rural dwelling [13, 15, 24]. The higher prevalence of anemia among pregnant women residing in rural areas may be due to inaccessibility to health care centers, lack of anemia-causing factors information, and inappropriate dietary habits.

In this study, anemia was preponderant among unemployed housewives (93.8%). This finding is inconsistent with studies conducted in Ethiopia [25], Uganda [26], and Pakistan [27]. Financial constraints, illiteracy, and not having early access to health care services may play an important role. In conflict-affected zones, social and financial barriers can act as major obstacles for women in seeking care.

Pregnant women of advanced age showed a higher risk of anemia in this study. This result is in agreement with findings from Ethiopia [28], Tanzania [29], and China [30]. However, contradictory findings are reported in studies from Mexico [31], Tabas [32], and Malawi [33]. Anemia in pregnancy is widely believed to increase with parity and maternal age.

This study found no significant association between anemia and monthly income, level of maternal education, and birth interval. In previous studies, however, a significant association was reported [13–18, 21, 33]. Differences in findings of previous reports and this study may be due to differences in monthly income and educational levels of study participants.

### 4.1. Limitations and Strengths

Firstly, this study was an institutional-based one. Therefore, the results of this study may not reflect what is going on at the level of the community. Secondly, some predictors of anemia were not deeply investigated. Thus, many considerations need to be taken into account in future research, such as antenatal care, history of bleeding, iron/folic acid supplementation, food security, dietary diversity, and marital age. Although this study has been an institutional-based one, which restricts its generalizability, it is the first study of its kind from Afghanistan populations.

## 5. Conclusion

In this study, the prevalence of anemia among pregnant women was 51 percent, which indicates a severe public health problem in the study area. Multiparity and no previous use of contraceptives were the independent factors that could significantly predict anemia among pregnant women. Hence, Afghanistan's policymakers must accelerate interventions to promote family planning in the country to reduce the prevalence of anemia among pregnant women. The results also suggest the need for prospective studies to identify other factors associated with anemia among pregnant women.

## Figures and Tables

**Table 1:** Socioeconomic and demographic characteristics of the pregnant women visiting Bost Hospital for delivery in Helmand province, 2019 (*n* = 787).

Characteristic	No. of cases	Percentage
Age group (Years)		
1. < 20	29	3.7
2. 20–24	131	16.6
3. 25–29	144	18.3
4. 30–34	215	27.3
5. 35–39	178	22.6
6. >40	90	11.4
Type of residence		
1. Urban	280	35.6
2. Rural	507	64.4
Maternal education		
1. Illiterate	391	53.3
2. Can read and write	177	24.1
3. Primary	77	10.5
4. Secondary (9–12)	41	5.5
5. Baccalaureate and above	47	6.4
Occupation status		
1. Housewife	738	93.8
2. Employed	47	6.2
Husband education		
1. Educated	301	38.2
2. Uneducated	407	51.7
Household monthly income (in Afghanis)		
1. <5000	26	3.3
2. 5000–10000	424	53.9
3. >10000	334	42.4
4. Don't disclose	3	0.4
Family size		
1. <5	85	10.8
2. >5	702	89.2

**Table 2:** Obstetrics and medical-related characteristics of the pregnant women visiting Bost Hospital for delivery in Helmand province, 2019 (*n* = 787).

Characteristic	No. of cases	Percentage (%)
Parity		
1. Single	701	89.1
2. Multiple	86	10.9
Previous use of contraceptive		
1. Yes	235	29.9
2. No	552	70.1
Birth interval		
1. <2 years	44	5.6
2. > 2 years	9	1.1
3. No child	133	16.6
4. Don't disclose	601	76.3

**Table 3:** Factors associated with anemia among pregnant women attending Bost Hospital for delivery in Helmand province, 2019: unadjusted and adjusted odds ratio.

Independent variable		Unadjusted odds ratio (95% CI)	*p* value	Adjusted odds ratio (95% CI)	*p* value
Age	<30	1	0.002	1	0.336
	≥30	1.19		1.18 (0.83–1.67)	
Residency	Urban	1	0.002	1	0.317
	Rural	1.71 (1.09–2.45)		1.18 (0.85–1.64)	
Occupation	Employed	1	0.017	1	0.019
	Unemployed	1.04		0.47 (0.25–0.88)	
Parity	Single	1	<0.001	1	<0.001
	Multiple	2.80 (2.34–3.51)		3.09 (1.81–5.29)	
Previous use of contraceptive	Yes	1	0.002	1	0.015
	No	1.15 (1.09–1.45)		1.53 (1.08–2.16)	

## Data Availability

The dataset is available and will be presented on request.
